# Unhealthy Immigrants: Sources for Health Gaps Between Immigrants and Natives in Israel

**DOI:** 10.3389/fsoc.2021.686306

**Published:** 2021-11-01

**Authors:** Keren Semyonov-Tal, Dina Maskileyson

**Affiliations:** ^1^ Department of Labor Studies, Tel Aviv University, Tel Aviv-Yafo, Israel; ^2^ Faculty of Management, Economics and Social Sciences, the Institute of Sociology and Social Psychology, University of Cologne, Cologne, Germany

**Keywords:** severity illness index, immigrants, nativity illness gap, Israel, decomposition analysis

## Abstract

The study focuses on sources for health gaps between Jewish immigrants and native-born Israelis. Unlike traditional immigrant societies where immigration is viewed as economically motivated, immigrants returning to Israel are viewed as the “returning diaspora”. Because immigrants in Israel are entitled to the same health benefits and medical services as native-born, we expect Israel to attract unhealthy immigrants in disproportionate numbers. The data for the analysis are obtained from the Israeli National Health Interview Survey (2013–2015). The data set provides detailed information on health status and illness, sociodemographic attributes and origin of immigrants. Three major origin groups of immigrants are distinguished: the former Soviet Union, Western Europeans or the Americans (mostly Ashkenazim), and Asians or North Africans (mostly Sephardim). Our findings lend support to the expectations that the health status of all immigrant groups is poorer than that of native-born Israelis. The nativity–illness gap is most pronounced in the case of male immigrants (from Europe or the Americas or South Africa or Australia) and for female immigrants (from countries in the Middle East or North Africa) and least pronounced in the case of immigrants arriving from the former Soviet Union for both gender groups. Decomposition of the gaps into components reveals that some portion of the illness gap can be attributed to nativity status, but the largest portion of the gap is attributed to demographic characteristics. Neither socioeconomic status nor health-related behavior accounts for a substantial portion of the nativity–illness gap for all subgroups of immigrants.

## Introduction

Scholars of international migration and health have repeatedly demonstrated that immigrants in traditional immigrant societies, such as the United States, Canada and Australia, tend to be healthier than comparable native-born populations (e.g., Donovan et al., 1992; McDonald and Kennedy, 2004; Argeseanu Cunningham et al., 2008). They also have demonstrated that with increasing length of stay in the host country, immigrants health tends to deteriorate and becomes similar to that of native born with similar characteristics (e.g., McDonald and Kennedy, 2004). This phenomenon is referred to in the literature as “the healthy immigrant effect” thesis. The logic embodied in the thesis contends that only healthy persons are likely to select themselves into new destinations where they have to compete for economic success. They do so despite limited access to health services and expensive medical treatment. Indeed, due to such selection processes, economic immigrants are expected to be healthier than the comparable native-born population and their health is expected to deteriorate with an increasing length of stay (e.g., Kreft and Doblhammer 2012).

In recent years, additional new models of immigration and immigrants health have been proposed; models that are not necessarily in line with the logic of the classic “healthy immigrant effect” model. Constant (2021) thoroughly discusses various additional models of migration such as “return migration” or “circular migration”. These models distinguish between emigration and remigration and the migration direction, which can have important effects on the motives and outcomes of international mobility as well as on immigrant’s health (e.g., Constant, 2021). However, in countries such as Israel where immigration is considered to be a “returning of Diaspora” rather than an economically motivated migration (e.g., Semyonov and Lewin-Epstein, 2003), the prevalence of the healthy immigrant effect or models such as “return migration” is questionable, if not highly doubtful (e.g., Constant et al., 2018). Although Jewish immigrants are (allegedly) “returning” to their homeland, in practice they are first-generation immigrants who are, unlike immigrants in other countries, entitled to all the civil rights and benefits equal to those of the native-born Israelis, including access to all health services. Unlike other immigrant societies, Israel opens its gates to Jewish immigrants regardless of their socioeconomic attributes or health criteria. The state grants citizenship and civil rights to the Jewish immigrants, including the rights to medical services and healthcare coverage upon arrival. That is, all residents (including new immigrants) are covered by the national health insurance and are entitled to health services that are provided to all residents.^1^ Indeed, in such a context of migration, one possible assumption is that a disproportional number of “less healthy” or “unhealthy” immigrants would be attracted to the new homeland, especially if they are emigrating from a country with a less accessible health care (for European examples, see Maskileyson et al., 2019).

The few studies that examined the health status of immigrants in Israel support the expectation that immigrants are not as healthy as the comparable native-born population (e.g., Baron-Epel and Kaplan, 2001; Constant et al., 2018). Yet it is important to note that the immigrant population of Israel is highly heterogeneous, with immigrants arriving from a wide variety of countries. In fact, Jewish immigrants arrive in Israel from practically every corner of the world. Whereas some arrive from highly developed, rich countries, such as North America and Western Europe, others come from the less economically developed countries of Asia and Africa and many arrive from Latin America as well as from countries of the former Soviet Union (e.g., Maskileyson, 2014). The motivation and reasons for migration and the socioeconomic characteristics, as well as the health status, may vary considerably across subgroups of immigrants. Likewise, the sources of health disparities between native-born Israelis and immigrants may vary. Therefore, it is far from clear whether and to what extent the “healthy immigrant effect” phenomenon or immigrants health advantage is present among the various groups of immigrants in Israel. It seems that Israeli immigration does not follow the rules of the classic “healthy immigrant effect” model or the alternative model of “returning immigrant”, but constitutes a test case of its own. Due to unavailability of data, it is difficult to test the thesis that immigrants to Israel are not as healthy as the comparable native-born Israelis at their time of arrival. Nevertheless, the available data enable us to test the argument that the immigrant populations in Israel are not as healthy as the comparable Jewish population even after a long stay in the country. The data also enable us to estimate the sources that are responsible for the nativity health gap in the context of Israeli society.

Therefore, in this study, we do not seek to focus on the healthy immigrant phenomenon or on changes in health of immigrants as compared to the native population over time or across generations but rather to contribute to the literature on sources for health disparities between immigrants and native-born. We do so by focusing on health disparities between immigrants and native-born in the context of the “returning diaspora” model of Israeli society. First, we examine the question whether and to what extent the immigrant health advantage is present across different nativity subgroups (i.e., immigrants of different origin and Israeli natives). We then further examine whether the sources of health gaps differ across subgroups of immigrants. To do so, we take advantage of a data set from the Israel National Health Interview Survey, INHIS-3 (2013–2015). The study population includes 4,511 Israelis aged 21 and over and a detailed series of self-reported information on illness and chronic conditions as well as on sociodemographic attributes of all respondents and country of origin of the immigrants. Such a data set provides us with a unique opportunity to examine the size and the sources of health gaps between native born and subgroups of immigrants in Israel. To the best of our knowledge, such an analysis has not been carried out in Israel or in other countries.

## Previous Research

### Immigrant Health Selection

The literature on the health of immigrants in traditional immigrant societies such as the United States, Canada and Australia provided firm support for the healthy immigrant effect thesis with studies arriving at the following findings: First, immigrants tend to be healthier, on average, than the comparable native population. Second, with passage of time in the host country, the health of immigrants tends to deteriorate and converge with the health level of the native citizens. Notably, this study does not aim to investigate the healthy immigrant effect as a whole; rather it focuses on the average health differences between immigrants and natives in Israel in general and for several subgroups of immigrants in particular. It also focuses on the sources for health disparities between the immigrant and native-born populations.

Immigrants better health is attributed, first and foremost, to positive health selection into immigration (e.g., Ronellenfitsch and Razum, 2004; Akresh and Frank, 2008). According to the literature, immigrants are likely to be originated from the healthier segments of the population and therefore more likely to be healthier than the residents of the host country (e.g., Abraído-Lanza et al., 1999; Palloni and Arias, 2004). Studies distinguish between two types of positive immigrant health selection. The first is individual self-selection, whereby potential immigrants are likely to be physically and mentally healthy because only healthy persons are capable of migration and are willing to confront the risks of migration. The second selection is imposed by the authorities of the destination country, who apply health screening procedures to potential immigrants (e.g., McDonald and Kennedy, 2004).

The results of a few studies also indicate the existence of a negative health selection of the ill and elderly, who emigrate to destinations of higher quality health care (e.g., Jasso et al., 2004; Maskileyson et al., 2019). It seems reasonable to expect that while expensive and limited access to medical services is likely to deter immigration of the unhealthy, generous welfare and health policies may be an important consideration for immigration and particularly attractive for unhealthy immigrants (e.g., Borjas, 1999; Jasso et al., 2004). According to Borjas (1999), welfare programs attract immigrants who would not have immigrated without these programs (i.e., “the welfare magnet”). This assumption is especially relevant for Israel—a country where selection and admission of immigrants is based on common ancestry and heritage (i.e., Jewishness) and where health status criteria are irrelevant and where immigrants are entitled to health and medical services upon arrival.

### Sources for Health Disparities Between Immigrants and Natives

The most common explanation for health disparities in the population is quite straightforward and is based on the role played by economic resources (e.g., Adams, 2003; Williams and Collins, 2016). According to this view, persons of lower economic status (e.g., immigrants, ethnic minorities and poor people) have limited access to advanced medical treatment and facilities or are unable to the purchase expensive medications due to the lack of economic resources (Semyonov et al., 2013; Semyonov et al., 2015). This line of explanation, however, is less relevant in a country such as Israel, where comprehensive public health is guaranteed to all residents.

Notably, despite the existence of an equitable “health basket” fund for all Israeli citizens, it obliges everyone to pay deductibles both for doctor visits and for prescription medications. The cost of certain medical drugs, for example, can be very costly and difficult to afford leading to the under-treatment of illnesses especially among disadvantaged populations. Although in Israel, public health insurance covers all citizens by the law (e.g., National Health Insurance Law, 1994), the residents may purchase extra health coverage, creating an inequality in access to health care. Indeed, differential rates of health insurance coverage can constitute one of the barriers experienced by disadvantaged populations in the access to higher quality medical services (e.g., Ku and Matani, 2001; Zuvekas and Taliaferro, 2003). Previous research clearly shows that in the United States, for example, ethnic and racial minorities and poorer people are less likely to purchase health insurance as compared to members of the majority population and wealthy people (e.g., Monheit et al., 2000; Semyonov et al., 2011).

Disparities in health can be also attributed to gaps in health care delivery based on age, gender and ethnicity (e.g., LaVeist, 2005; Saabneh, 2015). In the case of immigrants, disparities can also result from language barriers. That is, lack of language proficiency among immigrants may lead to underreporting of health problems and the inability to communicate, fill out medical forms and follow medical guidelines (e.g., Padela and Punekar, 2009). In addition, culturally influenced gender roles, norms, values, administrative barriers, bureaucracy as well as place of residence all can influence effective use of public health services and, thus, can contribute to health disparities between immigrants and native-born citizens (e.g., Feikin et al., 2009; Williams and Collins, 2016).

### The Israeli Context

Israel, unlike many immigration countries, opens its gates to every person of Jewish descent who wishes to immigrate to Israel. According to the “Law of Return, 1950” - a central feature of Israeli immigration law - people with Jewish ancestry can immigrate to Israel and obtain Israeli citizenship upon arrival (e.g., *Introduction*, “The Israeli Law of Return, 1950”^2^). In terms of welfare laws, Israeli citizenship grants full and equal access to education and public health as well as social security benefits and public housing. Immediately upon arrival, immigrants are entitled to the same welfare basket as every citizen of the State of Israel, including full access to all welfare and public health services. In this sense, the state of Israel does not select Jewish immigrants due to poor medical conditions. Previous studies reveal that in comparison to Israel-born natives, immigrants to Israel are more likely to report higher rates of ischemic heart disease, diabetes, hypertension and other chronic illnesses (e.g., Constant et al., 2018).

However, Israeli immigrants are not a homogeneous population because they arrive from a wide variety of countries. Therefore, they differ in their characteristics (e.g., Semyonov et al., 2015). For example, immigrants from the former Soviet Union were more likely to be economically active than other groups of immigrants, while immigrants from Europe and America were found to have better access to high-status lucrative jobs than immigrants from the former Soviet Union or Asia, Africa and Ethiopia (e.g., Semyonov et al., 2013). Immigrants from the former Soviet Union reported higher rates of disease and lower health indicators than Israeli-born residents (e.g., Baron-Epel, 2001). Davidovitch et al. (2013) concluded that economic and cultural factors influence health care utilization among immigrants and lead to inequality in health care delivery and health outcomes.

In the following, we examine health gaps by nativity status (i.e., across immigrants from different countries of origin) for both gender groups, respectively, and based on this information, we delineate the sources for the health disparities between immigrants and native-born Israelis. Specifically, we focus on Jewish immigrants from the former Soviet Union countries (hereafter FSU), Jewish immigrants of European origin from either Western Europe or the Americas or South Africa or Australia (hereafter EUAM), and Jewish immigrants from Middle Eastern countries and North Africa (hereafter MENA). We expect that health status of immigrants arriving from different countries of birth is likely to differ and that it will be lower than that of Israeli natives.

## Data Source and Variables

### Data

The data were obtained from the third Israel National Health Survey (INHIS-3) conducted in 2013–2015 by the National Center for Disease Control. The INHIS-3 is a cross-sectional, population-based survey, conducted by means of telephone interviews with a representative sample of the adult population dwelling in Israel (aged 21 and over). Random sampling of household telephone numbers was achieved via “DATARINGS” software, which contains data on landline telephone line subscribers in Israel. The response rate was 38.2% of contacted people among the Jewish population.

Data were collected through phone interviews conducted by the Survey Unit of the Israel Center for Disease Control (ICDC). The study population included 4,406 Israelis aged 21 and over (between the years 2013–2015), and the interviews were conducted both in Hebrew and in Arabic^3^. The questionnaire includes demographic characteristics as well as series of self-reported information on illness and chronic conditions. Because immigrants are selected and admitted to Israel mainly on the basis of their Jewish heritage (according to the Law of Return, 1950), only people of Jewish ancestry (immigrants and natives) were included in the analysis.^4^ That is, we excluded from the sample people who identified themselves as: Arab Muslim, Arab Christian, Druze, Bedouim, Cherkes, Arab (religion not specified).

Prior to analysis, cases of missing data (*n* = 137) for the following variables were deleted listwise (illness index = 6, immigrant status = 1, country of origin = 5, age = 1, marital status = 4, number of children = 18, years of education = 60, employment status = 1; physical activity = 25; smoking = 42).

### Variables

Nativity status is defined by place of birth, distinguishing between foreign-born (i.e., immigrants) and native Israeli born respondents. The immigrant population is further divided into three major geo-cultural (ethnic) origins: immigrants from FSU, EUAM and MENA.

The severity illness index, as an indicator of health status, is the dependent variable in the current study and is measured using a detailed list of self-reported illnesses. Self-reported illness and physical limitations have been shown to be useful predictors of physical health trajectories and mortality (e.g., Wolff et al., 2002; Huisman et al., 2003). The index was based on the 20 following self-reported health problems: asthma, hypertension, high cholesterol, triglycerides, heart attack, angina, heart failure, other heart disease, stroke, lung disease, joint disease, osteoporosis, Crohn’s disease, colitis, cancer or malignancy, migraine, anxiety, depression, thyroid disorder, diabetes. We weighted the items by their severity using the Duke Severity of Illness Checklist (DUSOI) (e.g., Parkerson et al., 1993; Parkerson et al., 1996) (see details in Appendix 1). Health problems were rated according to level of severity and impact on overall health. DUSOI is based upon the clinical judgment of health care providers and was developed entirely in the primary care setting. The reliability and validity of the DUSOI has been established (e.g., Parkerson et al., 1996). We multiplied each item by its DUSOI severity score and then created a sum score index. Severity illness index values were then standardized to a percentile ranking scale on which individuals are ranked, each according to his or her relative health on a percentile illness ladder.

Following previous studies (e.g., Deaton, 2008), we included a series of sociodemographic variables as control variables: age of respondent (in years), marital status (married = 1; not married = 0), and number of children. We also selected measured indicators of *socioeconomic status* that are known to impact health (e.g., Eikemo et al., 2008), including years of education, employment status (employed = 1, unemployed or not in the labor force = 0), and total monthly net household income measured in four categories of income of dummy variables: 1) less than 8,000 NIS; 2) 8,000–12,000 NIS; 3) higher than 12,000 NIS; 4) Missing income. The second category, intermediate income, serves as a comparison category. In addition, we also included three groups of variables that capture *health-related behaviors.* First, we created a dummy variable to indicate whether a person is a current or former smoker (= 1) vs non-smokers (0). We distinguished between those who exercise (= 1) and do not exercise (= 0). Finally, we included a variable capturing the nutrition habits of the respondents. This variable was presented by a set of dummy variables: 1) Less than one vegetable/fruit portion per day (= 1), 2) one to three vegetable/fruit portions per day (= 0), 3) More than three vegetable/fruit portions per day (= 1), 4) Missing for vegetable/fruit consumption (= 1) (see Appendix two for the definitions of the variables).

## Methods

We analyzed the data in three main steps. In step 1, we present a descriptive overview of the health, demographics, socioeconomic attributes and health-related behaviors for all subgroups of immigrants and Israeli natives by gender. In step 2, we estimate a set of regression equations predicting the severity illness index as a function of nativity status controlling for individual characteristics of the respondents. In step 3, we provide a decomposition analysis of the illness gaps between each subgroup of immigrant and Israeli-born native respondents into components attributed to differences in the nativity status, demographic characteristics, socioeconomic status and health-related behaviors. Notably, Israeli-born natives were, on average, considerably healthier than immigrants (see Table 1). Assuming that there might be a selection issue, we performed selectivity bias adjustment. Specifically, we fit a regression model with sample selection in the following two steps: 1) we estimated the probit model for the sample selection equation predicting whether the person has reported having any health difficulties (vs being healthy). The explanatory variables included in the selection probit equation are the same as for the ordinary least squares (OLS) regression of the second step (age, marital status, number of children, years of education, employment status, income, fruit and vegetable consumption, physical activity and smoking). The inverse Mills ratio calculated on the basis of this probit model. The inverse Mills ratio corrects for potential bias in estimates due to selection (non-random assignment) into having any illness. 2) Using the selected sample, we fitted the second step OLS model by adding the inverse Mills ratio (or “non-selection hazard”) from the first step to the main OLS equation as an additional independent variable (e.g., Manning et al., 1987). The significance of the inverse Mills ratio is an indication of selection effects.

**TABLE 1:** Means and distributions of variables, by nativity status and gender.

	Men					Women				
	Israeli natives	All immigrants	FSU	EUAM	MENA	Israeli natives	All immigrants	FSU	EUAM	MENA
Health measures	—	—	—	—	—	—	—	—	—	—
Has any illness, %	64	77	65	82	80	59	82	76	84	86
Does not have any illnesses, %	36	23	35	18	20	41	18	24	16	14
Illness index (percentiles), mean (SD)	40.19	52.39	41.00	59.55	53.16	38.40	57.09	49.46	58.09	62.70
	(33.48)	(33.53)	(33.67)	(33.65)	(31.10)	(34.81)	(31.87)	(32.41)	(31.41)	(30.70)
Demographics	—	—	—	—	—	—	—	—	—	—
Years since migration	—	46.09	29.27	48.08	56.46	—	43.38	29.41	43.71	55.14
	—	(18.40)	(12.07)	(19.82)	(10.16)	—	(18.44)	(14.07)	(18.69)	(12.19)
Age (in years)	51.31	63.89	53.77	65.91	69.25	49.19	61.39	55.02	61.54	66.86
	(15.22)	(14.84)	(14.78)	(15.81)	(9.07)	(13.97)	(14.01)	(13.86)	(15.56)	(9.68)
Married, %	85	85	83	85	85	83	70	68	71	71
Not married, %	15	15	17	15	15	17	30	32	29	29
Number of children, mean (SD)	3.10	3.03	2.09	2.85	3.93	3.20	3.03	2.08	3.25	3.65
	(1.95)	(1.85)	(1.60)	(1.59)	(1.88)	(1.84)	(1.85)	(1.09)	(2.10)	(1.80)
Socioeconomic status	—	—	—	—	—	—	—	—	—	—
Years of education, mean (SD)	15.40	14.36	15.14	15.78	12.27	14.82	13.63	15.05	14.94	11.18
	(4.89)	(5.05)	(3.16)	(5.03)	(5.52)	(3.07)	(3.96)	(3.11)	(3.85)	(3.41)
Employed, %	70	45	70	39	32	70	39	62	38	19
Unemployed or out of the labor force, %	30	55	30	61	68	30	61	38	62	81
Monthly household net income less than 8,000 NIS, %	18	28	19	18	45	25	40	39	28	54
Monthly household net income 8,000–12,000 NIS, %	22	24	31	21	23	23	21	25	21	17
Monthly household net income higher than 12,000 NIS, %	45	34	38	44	20	36	20	26	28	8
Missing income, %	16	14	11	18	13	16	18	10	23	21
	—	—	—	—	—	—	—	—	—	—
Current or former smoker, %	49	60	62	59	61	31	36	36	34	39
Never smoked, %	51	40	38	41	39	69	64	64	66	61
Participates in physical activity/sports, %	67	61	59	66	57	58	63	59	69	59
Does not participate in physical activity/sports, %	33	39	41	34	43	42	37	41	31	41
Less than 1 vegetable/fruit portion per day, %	27	22	21	21	23	23	19	17	15	26
1–3 vegetable/fruit portions per day, %	60	63	61	67	61	67	69	68	76	63
More than 3 vegetable/fruit portions per day, %	9	9	12	8	8	8	8	11	8	4
Missing for vegetable/fruits, %	4	6	7	4	8	3	4	4	2	7
*Observations*	875	606	160	231	215	903	530	160	185	185

Note: mean coefficients; SD in parentheses.

Except for the proportion of ill people, all calculations are made for the sample of respondents who have reported having at least one illness.

We applied the Oaxaca (1973) and Blinder (1973) decomposition procedure to separate between different sources of the nativity–illness gaps. Notably, while this decomposition method has mostly been applied to wage and income inequality (e.g., Fortin et al., 2011), it can be used to understand the sources of health inequality (e.g., O’Donnell et al., 2006; O’Donnell et al., 2012). To estimate the illness gap, we decomposed the mean difference between the immigrant groups via the use of linear regression models for males and females separately (e.g., Maskileyson et al., 2021). This allowed us to distinguish between two components: 1) a component of the illness gap that is explained by the differences in individual attributes, such as demographics, socioeconomic status, health-related behavior (the Xs); and 2) the unexplained component of the illness gap attributed to unmeasured characteristics (the βs). To account for selection bias in the decomposition analysis, similarly to the regression analysis described above, we estimated the probit model and then applied the standard Oaxaca decomposition formulas adding the inverse Mills ratio from the first step (e.g., Neuman and Oaxaca, 2004).

The decomposition is performed according to the following notation:

$${\bar{Y}}_{in}-{\bar{Y}}_{im}=\sum \left({\bar{x}}_{in}-{\bar{x}}_{im}\right)\beta_{\rho }+\left[\sum {\bar{x}}_{im}\left(\beta_{\rho }-\beta_{im}\right)+{\bar{x}}_{in}\left(\beta_{\rho }-\beta_{in}+\left(\alpha_{in}-\alpha_{im}\right)\right)\right]$$

where ${\bar{Y}}_{in}$ and ${\bar{Y}}_{im}$ are severity illness indices of Israeli natives and immigrants, respectively. For the sake of parsimony, we refer here generally to immigrants, but we compared each subgroup of immigrants (i.e., FSU, EUAM and MENA) to Israeli natives. ${\bar{x}}_{in}$ and ${\bar{x}}_{im}$ are means of all predictors, and $\beta_{in}$ and $\beta_{im}$ are the coefficients of these predictors for Israeli natives and immigrants, respectively. $\beta_{\rho }$ are the coefficients from a pooled regression. $\sum \left({\bar{x}}_{in}-{\bar{x}}_{im}\right)\beta_{\rho }$ is the portion of the gap that is explained by nativity differences in mean illness attributes. $\left[\sum {\bar{x}}_{im}\left(\beta_{\rho }-\beta_{im}\right)+{\bar{x}}_{in}\left(\beta_{\rho }-\beta_{in}+\left(\alpha_{in}-\alpha_{im}\right)\right)\right]$ reflects the portion of the gap attributed to differences in individual attributes.

## Results

### Descriptive Overview

Table 1 presents a descriptive overview of health in terms of percentage of people who have reported having at least one health problem and illness index (mean) on the percentile 100-point scale, for Israeli natives and for all subgroups of immigrants, by gender. Also, in Table 1 we report the socioeconomic, sociodemographic and health-related behavior mean differences of immigrants and the native-born population. The data presented in Table 1 reveal that all subgroups of immigrants are more likely to report poorer health as compared to Israeli natives. While 77% of immigrant men and 82% of immigrant women have reported at least one health problem, only 64% of Israeli men and 59% of Israeli women did so. Mean illness index of all subgroups of immigrants, without exception, was also higher than that of the Israeli native population of both genders. The health of immigrants from EUAM or MENA was considerably poorer than the health of the FSU immigrants. These patterns hold within both gender groups.

Immigrants differ from the native-born population not only in levels of health but also with respect to an array of socioeconomic and demographic characteristics. Immigrants also differ from Israeli native-born in their health behavior patterns. Immigrants tend to be 10 years older, on average, than the Israeli native-born population with FSU immigrants the youngest among the three immigrant subgroups. While the share of married people somewhat varies only among women, there are considerable differences in average number of children among all subgroups, with highest number of children among immigrants from MENA countries. Differences in educational level between immigrants and natives also vary by country of origin. The average number of years of education of immigrants from MENA countries is lower than that of native-born Israeli citizens. The educational level of FSU and EUAM immigrants is similar or even higher than that of the natives. The employment rates of Israeli native men and women are considerably higher than that of immigrants (with an exception of FSU immigrants), which is related to the younger age of the natives. Immigrants from all countries, without exception, tend to have considerably lower income as compared to natives. When it comes to health-related behavior, Israeli natives tend to smoke less, exercise more but have a somewhat less healthy diet as compared to immigrants.

### Multivariate Analysis of Illness by Nativity Status

While the descriptive results revealed interesting differences among immigrants and natives, it is not clear whether and to what extent differences in health between the Israeli native population and subgroups of immigrants can be attributed to immigrant status, place of origin, to differences in sociodemographic or socioeconomic attributes of the immigrants or differences in health-related behavior. Therefore, in the analysis that follows, we estimate a series of regression models predicting illness index (presented as percentile 100-point scale). We conduct the two-step model including a separate probit model for sample selection bias followed by an OLS regression (e.g., Manning et al., 1987). Dichotomous variable—having an illness vs being healthy—serves as a dependent variable for the probit model. The probit model allowed us to estimate Mills ratios (introduced in the OLS regression to correct for selectivity bias)^5^.

Tables 2, 3 display the coefficients of eight OLS regression equations predicting the illness index for men and women, respectively. In Equations 1a–4a, immigrant status is defined by a dummy variable distinguishing between immigrants and Israeli natives. In Equations 1a–4b, immigrant status is defined by three dummy variables representing immigrant’s origin (i.e., FSU, EUAM and MENA) versus Israeli native-born population. In Equations 1a,b, we let illness index be a function of immigrant status. In Equations 2a,b, 3a,b, we add demographics and socioeconomic attributes, respectively, as predictors of the illness index. In Equations 4a,b, we add health-related behaviors to the set of predictors of the illness status. All models also include inverse Mills ratio, to account for a possible selection bias.

**TABLE 2:** Coefficients (standard errors) of OLS regression equations predicting severity illness index, Men.

Variables	Model 1a	Model 2a	Model 3a	Model 4a	Model 1b	Model 2b	Model 3b	Model 4b
Immigrant group	—	—	—	—	—	—	—	—
Immigrant (ref. Israeli natives)	−0.01	−0.43	−0.86	−1.05	—	—	—	—
	(1.70)	(1.71)	(1.73)	(1.73)	—	—	—	—
FSU (ref. Israeli natives)	—	—	—	—	−2.44	−2.86	−2.89	−3.01
	—	—	—	—	(2.56)	(2.60)	(2.61)	(2.63)
EUAM (ref. Israeli natives)	—	—	—	—	5.40*	4.92*	4.88*	4.36
	—	—	—	—	(2.32)	(2.34)	(2.37)	(2.38)
MENA (ref. Israeli natives)	—	—	—	—	−3.83	−4.02	−5.61*	−5.47*
	—	—	—	—	(2.43)	(2.48)	(2.56)	(2.56)
Demographics	—	—	—	—	—	—	—	—
Age (in years)	—	0.15	0.16	0.55*	—	0.12	0.11	0.42
	—	(0.16)	(0.16)	(0.24)	—	(0.16)	(0.16)	(0.24)
Married (ref. not married)	—	−2.30	−0.66	0.76	—	−2.65	−0.78	0.34
	—	(2.42)	(2.60)	(2.69)	—	(2.42)	(2.59)	(2.69)
Number of children	—	−0.54	−0.64	−1.00	—	−0.39	−0.39	−0.69
	—	(0.47)	(0.49)	(0.53)	—	(0.48)	(0.51)	(0.54)
Socioeconomic status	—	—	—	—	—	—	—	—
Years of education	—	—	−0.11	−0.01	—	—	−0.23	−0.13
	—	—	(0.17)	(0.17)	—	—	(0.17)	(0.18)
Employed (ref. unemployed or not in the labor force)	—	—	−3.40	−4.55*	—	—	−3.13	−4.05*
	—	—	(1.95)	(2.02)	—	—	(1.96)	(2.03)
Income less than 8,000 NIS (ref. 8,000–12,000 NIS)	—	—	0.60	2.26	—	—	1.10	2.41
	—	—	(2.45)	(2.55)	—	—	(2.45)	(2.55)
Income higher than 12,000 NIS (ref. 8,000–12,000 NIS)	—	—	−0.69	0.46	—	—	−1.27	−0.28
	—	—	(2.10)	(2.15)	—	—	(2.10)	(2.16)
Missing income (ref. 8,000–12,000 NIS)	—	—	−1.33	0.25	—	—	−1.78	−0.48
	—	—	(2.60)	(2.69)	—	—	(2.60)	(2.69)
Health behavior	—	—	—	—	—	—	—	—
Less than 1 vegetable/fruit portion per day (ref. 1–3 vegetable/fruit portions per day)	—	—	—	0.44	—	—	—	0.09
	—	—	—	(1.97)	—	—	—	(1.97)
More than 3 vegetable/fruit portions per day (ref. 1–3 vegetable/fruit portions per day)	—	—	—	−2.46	—	—	—	−1.94
	—	—	—	(2.91)	—	—	—	(2.91)
Missing for vegetable/fruits (ref. 1–3 vegetable/fruit portions per day)	—	—	—	−6.07	—	—	—	−4.15
	—	—	—	(4.60)	—	—	—	(4.62)
Current or former smoker (ref. never smoked)	—	—	—	5.27**	—	—	—	4.71*
	—	—	—	(1.91)	—	—	—	(1.91)
Does any sport (ref. does not do sport)	—	—	—	1.49	—	—	—	1.19
	—	—	—	(1.77)	—	—	—	(1.77)
Mills	−51.45**	−45.15**	−41.74**	−19.44	−51.16**	−46.10**	−43.69**	−25.82**
	(2.62)	(7.93)	(8.48)	(12.80)	(2.69)	(7.92)	(8.46)	(12.90)
Constant	70.49**	62.77**	63.79**	25.56	70.32**	64.54**	68.49**	37.59
	(1.85)	(12.76)	(14.22)	(21.73)	(1.88)	(12.73)	(14.22)	(21.96)
	—	—	—	—	—	—	—	—
Observations	1,481	1,481	1,481	1,481	1,481	1,481	1,481	1,481
R-squared	0.23	0.23	0.24	0.24	0.24	0.24	0.24	0.25
Adjusted R-squared	0.230	0.231	0.231	0.232	0.235	0.236	0.237	0.237

Note: Standard errors in parentheses; ** p < 0.01, * p < 0.05; All models are estimated based on a two-stage estimation procedure using the inverse Mills ratio to correct for the selection bias.

**TABLE 3:** Coefficients (standard error) of OLS regression equations predicting severity illness index, Women.

Variables	Model 1a	Model 2a	Model 3a	Model 4a	Model 1b	Model 2b	Model 3b	Model 4b
Immigrant group	—	—	—	—	—	—	—	—
Immigrant (ref. Israeli natives)	4.78**	4.31*	3.18	3.40	—	—	—	—
	(1.75)	(1.76)	(1.77)	(1.77)	—	—	—	—
FSU (ref. Israeli natives)	—	—	—	—	3.39	2.64	1.94	2.28
	—	—	—	—	(2.56)	(2.60)	(2.61)	(2.61)
EUAM (ref. Israeli natives)	—	—	—	—	6.36*	5.89*	5.55*	5.73*
	—	—	—	—	(2.47)	(2.49)	(2.49)	(2.50)
MENA (ref. Israeli natives)	—	—	—	—	4.49	4.36	1.74	1.83
	—	—	—	—	(2.57)	(2.60)	(2.70)	(2.70)
Demographics	—	—	—	—	—	—	—	—
Age (in years)	—	0.29	0.34	0.64*	—	0.28	0.33	0.60*
	—	(0.18)	(0.19)	(0.28)	—	(0.18)	(0.19)	(0.28)
Married (ref. not married)	—	−2.06	0.61	2.17	—	−2.07	0.70	2.14
	—	(2.10)	(2.22)	(2.43)	—	(2.10)	(2.22)	(2.43)
Number of children	—	−0.52	−1.01*	−1.25*	—	−0.58	−1.04*	−1.23*
	—	(0.49)	(0.50)	(0.56)	—	(0.50)	(0.51)	(0.57)
Socioeconomic status	—	—	—	—	—	—	—	—
Years of education	—	—	−0.09	0.12	—		−0.15	0.05
	—	—	(0.26)	(0.26)	—	—	(0.27)	(0.27)
Employed (ref. unemployed or not in the labor force)	—	—	−5.22*	−6.56**	—	—	−5.04*	−6.29**
	—	—	(2.19)	(2.34)	—	—	(2.20)	(2.35)
Income less than 8,000 NIS (ref. 8,000–12,000 NIS)	—	—	2.83	3.80	—	—	2.94	3.83
	—	—	(2.34)	(2.43)	—	—	(2.34)	(2.43)
Income higher than 12,000 NIS (ref. 8,000–12,000 NIS)	—	—	−3.30	−2.45	—	—	−3.40	−2.60
	—	—	(2.31)	(2.35)	—	—	(2.31)	(2.36)
Missing income (ref. 8,000–12,000 NIS)	—	—	−2.73	−1.90	—	—	−2.92	−2.17
	—	—	(2.62)	(2.69)	—	—	(2.63)	(2.70)
Health behavior	—	—	—	—	—	—	—	—
Less than 1 vegetable/fruit portion per day (ref. 1–3 vegetable/fruit portions per day)	—	—	—	2.94	—	—	—	2.92
	—	—	—	(2.11)	—	—	—	(2.11)
More than 3 vegetable/fruit portions per day (ref. 1–3 vegetable/fruit portions per day)	—	—	—	−2.56	—	—	—	−2.36
	—	—	—	(3.06)	—	—	—	(3.07)
Missing for vegetable/fruits (ref. 1–3 vegetable/fruit portions per day)	—	—	—	−3.87	—	—	—	−3.13
	—	—	—	(5.43)	—	—	—	(5.46)
Current or former smoker (ref. never smoked)	—	—	—	3.49	—	—	—	3.32
	—	—	—	(2.09)	—	—	—	(2.09)
Does any sport (ref. does not do sport)	—	—	—	−3.46^*^	—	—	—	−3.58*
	—	—	—	(1.72)	—	—	—	(1.73)
Mills	−54.85**	−41.51**	−34.09**	−19.06	−54.72**	−41.54**	−34.87**	−21.28
	(2.63)	(8.30)	(9.26)	(14.17)	(2.67)	(8.32)	(9.27)	(14.27)
Constant	73.99**	54.68**	52.44**	24.98	73.91**	55.24**	54.45**	29.38
	(1.97)	(13.96)	(15.94)	(24.44)	(1.99)	(13.98)	(16.02)	(24.66)
	—	—	—	—	—	—	—	—
Observations	1,433	1,433	1,433	1,433	1,433	1,433	1,433	1,433
R-squared	0.28	0.29	0.30	0.30	0.29	0.29	0.30	0.30
Adjusted R-squared	0.284	0.285	0.292	0.296	0.283	0.285	0.292	0.296

Note: Standard errors in parentheses; ** p < 0.01, * p < 0.05; All models are estimated based on a two-stage estimation procedure using the inverse Mills ratio to correct for the selection bias.

The findings of the regression adjusted for a selection bias reveal that the average health of male immigrants does not significantly differ from their Israeli born counterparts (see Table 2). Similarly, no statistically significant difference is found between male immigrant subgroups and male Israeli natives. Only the EUAM male immigrants have a significantly higher illness level as compared to native-born Israelis; however, this disadvantage disappears after controlling for health-related behaviors. A somewhat different picture can be observed for women (Table 3). Models 1a-4a reveal that the health of female immigrants is significantly lower than the health of native-born Israeli women, even after controlling for individual attributes and health-related behaviors. The health disparity is most pronounced for the group of EUAM female immigrants [b = 5.73 percent in Eq. 4b]. Therefore, EUAM immigrant women are less healthy than Israeli natives with similar demographic and socioeconomic characteristics and similar health-related behavior. Health of FSU and MENA immigrants does not significantly differ from that of the natives, especially after controlling for variations in individual attributes.

Not surprisingly, the coefficients representing individual characteristics reveal that illness tends to increase with older age. A higher number of children is associated with better health. One possible explanation is that people who are physically capable to become parents are a selective group with better health metrics. Illness is strongly associated with smoking, leading to deterioration of health. Finally, when looking at the contribution of the Mills ratio to the within-groups variance of illness index, it becomes apparent that the coefficient of Mills ratio in the models 1a-3a and 1–3b for both genders is negative and statistically significant. Therefore, we can conclude that there are unobserved variables increasing the probability of selection and the probability of a lower-than-average score on the dependent variable. However, after including health-related behavior variables to models 4a-4b, Mills ratio becomes insignificant for both men and women (e.g., Lennox et al., 2012). This means that when health related behavior variables are included, selection bias disappears.

### Decomposing the Illness Gap by Nativity Status

In this section we decompose the illness gap between each subgroup of immigrants and Israeli native-born into two major components: 1) immigration status and ethnicity (unexplained component) and 2) differences in individual characteristics. The latter is further divided into mean differences of specific characteristics. For the sake of parsimony, we aggregated the coefficients into three distinct components: demographics (i.e., age, marital status, number of children), socioeconomic status (i.e., years of education, employment status and income), health-related behavior (i.e., smoking, sport, consumption of fruits and vegetables). The results of the decomposition analysis are presented in Appendix 3 for each subgroup of immigrants, by gender. The coefficients are presented in terms of the percentiles.

Examination of the immigrants illness gaps reveals that the largest illness gap is observed between Israeli-born males and EUAM male immigrants (38.7%) and between Israeli-born females and MENA female immigrants (49.7%). Among men, the adjusted illness gap between Israeli-born and FSU immigrants as well as between Israeli-born and MENA immigrants is not statistically significant. Among women, the illness gap between Israeli-born and FSU immigrants is not statistically significant and Israeli-born and EUAM immigrants is relatively large and equals about 44%.

**FIGURE 1:**
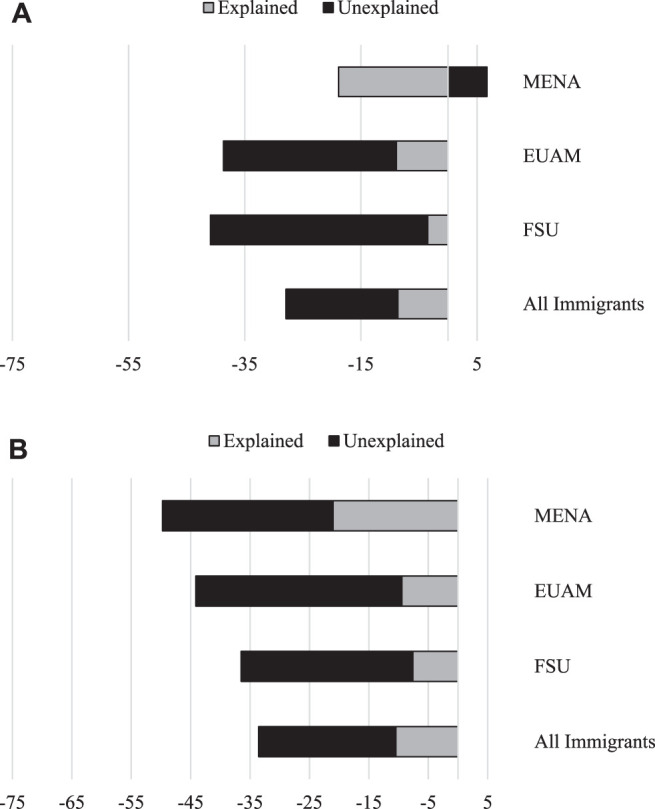
**(A)** Decomposition of the total gap in the severity illness index (measured on a 100-point percentile scale) by nativity status, Men. **(B)**: Decomposition of the total gap in the severity illness index (measured on a 100-point percentile scale) by nativity status, Women.

**FIGURE 2:**
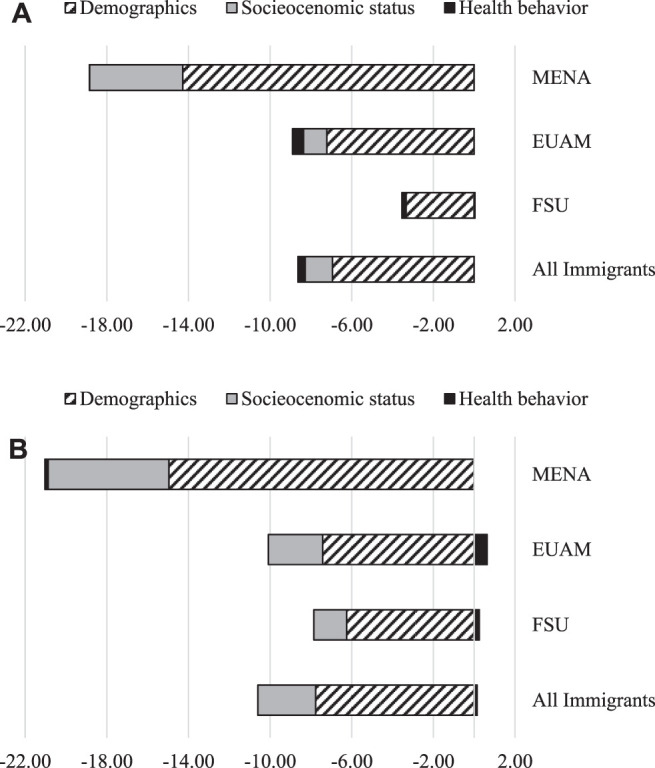
**(A)** Components of nativity illness gap, Men. **(B)**: Components of nativity illness gap, Women.

Figure 1A shows that among men, most of the gap between the Israeli-born and EUAM immigrant participants (23% out of total 38.6%) is attributed to nativity status and only 8.9% to the differences in characteristics of the individuals. Likewise, among women, most of the gap between the Israeli-born and EUAM immigrant participants (34.7% out of total 44.1%) and between the Israeli-born and MENA immigrant participants (28.7% out of total 49.7%) is attributed to the nativity status (Figure 1B). A graphic illustration of the specific sources of the gaps for each subgroup is provided in Figure 2A, B for men and women, respectively.

Figure 2A demonstrates that differences in demographic characteristics such as age, marital status and number of children are the most important determinants of the illness gaps across all immigrant male and female subgroups. Demographics account for about 3%, 7% and 14% of the illness gaps between Israeli-born men and FSU, EUAM and MENA immigrant men, respectively. Interestingly, neither socioeconomic characteristics nor health-related behavior account for much of the illness gap between the Israeli-born and immigrant subgroups. Differences in socioeconomic status account for a small share of the illness gap (4.6%) only between MENA male immigrants and their Israeli-born counterparts. Similarly for women, differences in demographic characteristics account for about 6%, 7% and 15% of the illness gaps between Israeli-born and FSU, EUAM and MENA immigrant women, respectively (see Figure 2B). Differences in socioeconomic status among women account for 2, 3, and 6% of the illness gap between FSU, EUAM and MENA female immigrants and their Israeli-born counterparts, respectively.

## Strength and Limitations

This study underscores the importance of mapping sources of health disparities between immigrants and native-born in the context of Israeli society. Despite its valuable contribution to knowledge, this study has several limitations, such as a possible selectivity effect regarding the years since migration, reasons for migration and the health status upon arrival at the destination country. Furthermore, the estimated model includes a selection of sociodemographic and socioeconomic variables that correlate with individual health, such as income, education, employment status, marital status and number of children. Due to lack of data, the analysis does not include such important variables as, for example, social inclusion (e.g., number of friends, feelings of social isolation).

Despite these limitations, the present study is one of the very few that investigates sources of health disparities between immigrants and native-born in general, and to the best of our knowledge the first one in the Israeli context. Indeed, the systematic and significant associations found throughout the analysis increase the confidence in the reliability of the findings and the contribution to knowledge.

## Discussion and Conclusion

Consistent with our expectation, we find gaps in illness indices between immigrants and Israeli natives, with natives being healthier than immigrants. The analysis clearly shows that, unlike other traditional immigrant societies, the health of all groups of immigrants is considerably poorer than that of Israeli natives, even after controlling for age. We suggest that this finding might result from a negative self-selection of immigrants with regard to health. Indeed, this finding underscores our initial argument that the classic model of the “healthy immigrant effect” does not prevail or apply in societies such as Israel and in the context of “returning diaspora” migration. Immigrants to Israel, unlike immigrants to other traditional immigration societies (such as the United States, Canada or Australia) cannot be viewed as economic immigrants who tend to be selected from the healthier segments of the population. Indeed, the selection process of immigrants to Israel differs starkly from selection of immigrants to other societies. That is, Jewish immigrants to Israel are selected only on the basis of ancestry regardless of their socioeconomic status or health. Furthermore, immigrants in Israel are covered by national health insurance and, therefore, are entitled to citizenship and medical services upon arrival to the country. Indeed, such a context is likely to attract many less healthy immigrants.

Using the decomposition method (O’Donnell et al., 2012), we compared health as defined by the severity illness index and examined the sources of the health disparities across different nativity groups by gender. Although all immigrant groups are characterized by poorer health than the comparable Israeli born, the findings reveal that the nativity–illness gap is most pronounced in the case of EUAM male immigrants (arriving from Europe, the Americas, South Africa, or Australia)^6^ and MENA female immigrants (from Middle Eastern countries and North Africa). By contrast, the nativity–illness gap is least pronounced in the case of immigrants arriving from FSU countries for both gender groups [partly contracting Baron-Epel’s (2001) findings]. Focusing on the sources for the health gaps, the data reveal that whereas some portion of the illness gap can be attributed to nativity status, the largest portion of the gap is attributed to demographic characteristics.

In addition, the data demonstrate that health of both immigrants to Israel and native-born Israelis tends to deteriorate with age and that married persons and those who are parents tend to be healthier than singles and non-parents. The analysis shows that differences in age, marital status and parenthood explain a substantial portion of the nativity–illness gap. This may be attributed to the importance of a supportive family network for health. It can be assumed that a larger family unit is able to provide more support and medical supervision, thereby assuring the well-being of family members. Immigrants are more likely to be socially isolated living apart from their extended family network (if some of their family members still reside abroad). Therefore, they tend to receive less family support (e.g., assistance with medical care, nursing, or financial aid) as compared to the native-born Israelis and, therefore, have poorer health.

As expected, the data also show that health-related behavior, such as exercise, is positively associated with health, while smoking exerts a negative effect on the health of both immigrants and natives. Nutrition (e.g., intake of fresh fruits and vegetables), on the other hand, does not have a statistically significant association with health.

Curiously, however, the decomposition analysis reveals that neither differences in socioeconomic status nor differences in health-related behavior account for a substantial portion of the nativity–illness gap for all subgroups of immigrants. The negligible (unexpected) effect of income on health and on health disparities between immigrants and natives can be attributed to the openness of the Israeli public health system, which provides mostly free access to health services to all residents regardless of their nativity status. Therefore, lack of financial capacity becomes less of a factor in explaining differential access to health services in Israeli society. In other words, the lower income of immigrants does not preclude them from access to medical services and medical treatment.

Yet despite the free access to medical services in Israeli society, a negative effect of the lowest income category on health status and health disparities is observed, implying that the health of the poorest people is worse than the health of all others. This finding can be attributed to the “deductibles” charged for treatments and to additional charges when purchasing specific medications. Paying such deductibles may still be burdensome for low-income individuals, who cannot finance exclusive treatments or cannot purchase specific medications. Likewise, low-income residents, despite their free access to public medical services, may not be able to purchase additional (private) health insurance which covers the use of private medicine. Lack of additional coverage may have, in turn, detrimental consequences for the health of the poor regardless of whether the poor citizens are immigrants or native born.

To summarize, this study examines the sources for illness gaps between three Jewish immigrant groups (EUAM, FSU, and MENA) and native-born Jewish Israelis. In line with previous studies (e.g., Constant et al., 2018), our findings reveal that the health status of all immigrant groups is poorer than that of native-born Israelis. The nativity–illness gap is most pronounced in the case of male EUAM immigrants and for female MENA immigrants and least pronounced in the case of immigrants arriving from the FSU for both gender groups. Decomposition of the gaps into components reveals that some portion of the illness gap can be attributed to nativity status, but the largest portion of the gap is attributed to demographic characteristics (i.e., age, marital status, number of children). Neither socioeconomic status nor health-related behavior accounts for a substantial portion of the nativity–illness gap for all subgroups of immigrants. While immigrant health selection is not directly measurable with the data at hand, we argue that it can be a part of the unexplained illness gap between immigrants and natives. That is, in the context of “returning diaspora” migration, unhealthier immigrants may be drawn to the health care system and social benefits.

## Data Availability

The raw data supporting the conclusions of this article will be made available by the authors, without undue reservation.

## Footnotes

1 Notably, all Jewish immigrants can automatically become citizens of Israel upon arrival and, therefore, are eligible to join a health fund and gain accessibility to public health services.

2 The State of Israel recognizes that every Jew (as well as his or her spouse, children, grandchildren, and their spouses) as having a nearly absolute claim to be admitted and to settle in Israel (*Introduction*, “The Israeli Law of Return, 1950”).

3 The interviews in Hebrew were aimed at the Jewish population, while the interviews in Arabic were aimed at the Arab population. However, it is possible that a sample of Jewish residents were interviewed in Arabic, for example Jews from African-Asian countries who at the time of the interview were not fluent in Hebrew or chose to be interviewed in Arabic (i.e., Hebrew is not their first language).

4 The INHIS-3 survey includes 4,406 Israeli residents from three population groups: 2,919 Jews, 1,347 Arabs (Muslims, Christians, Druze, and others living among the Arab population), and 140 others (i.e., non-Arab Christians and those who do not identify themselves with any religion). While the Israeli immigration policy encourages the migration of people of Jewish descent without imposing any restrictions, other immigrants (i.e., labor immigrants, asylum seekers, and refugees) have no (or unresolved) legal status (Nakash et al., 2012) and their share is relatively low as compared to other immigrant receiving countries. Using this survey, it was not possible to ascertain other immigrant groups (i.e., labor immigrants, asylum seekers, and refugees).

5 We do not include years since migration (YSM) in the analysis because in this study we do not focus on the healthy immigrant effect but rather on the health differences and their sources among the natives and immigrants at the time of the survey.

6 Over the 50% of our EUAM sample include immigrants from Eastern Europe and Germany, who are more likely to be Holocaust survivors or Holocaust survivors children. Literature shows that survivors tend to suffer lasting physical, mental, psychological, and social impairments. Their trauma recovery was also more difficult because the families and communities, through which they could gain support, were destroyed (e.g., Harel et al., 1993). Therefore, the historical background of EUAM group may serve as another explanation for their poorer health status.

## References

[B1] Abraído-Lanza A. F. Dohrenwend B. P. Ng-Mak D. S. Turner J. B. (1999). The Latino Mortality Paradox: a Test of the “Salmon Bias” and Healthy Migrant Hypotheses. Am. J. Public Health. 89 (10), 1543–1548. 10.2105/AJPH.89.10.1543 10511837 PMC1508801

[B2] Adams P. Hurd M. D. McFadden D. Merrill A. Ribeiro T. (2003). Healthy, Wealthy, and Wise? Tests for Direct Causal Paths between Health and Socioeconomic Status. J. Econom. 112 (1), 3–56. 10.1016/S0304-4076(02)00145-8

[B3] Akresh I. R. Frank R. (2008). Health Selection Among New Immigrants. Am. J. Public Health. 98 (11), 2058–2064. 10.2105/AJPH.2006.100974 18309141 PMC2636435

[B4] Argeseanu Cunningham S. Ruben J. D. Narayan K. M. (2008). Health of Foreign-Born People in the United States: a Review. Health Place. 14 (4), 623–635. 10.1016/j.healthplace.2007.12.002 18242116

[B5] Aryeh Greenfield-A.G Publication (1994). National Health Insurance Law, 5754-1994. Berkeley, California: Berkeley Law. Official Government Gazette (Codex 1469, June 26, 1994) (Hebrew).

[B6] Baron-Epel O. Kaplan G. (2001). Self-Reported Health Status of Immigrants From the Former Soviet Union in Israel. Isr. Med. Assoc. J. 3, 940–946. 11794920

[B7] Blinder A. S. (1973). Wage Discrimination: Reduced Form and Structural Estimates. J. Hum. Resour. 8 (4), 436–455. 10.2307/144855

[B8] Borjas G. J. (1999). Immigration and Welfare Magnets. J. Labor Econ. 17 (4), 607–637. 10.1086/209933

[B10] Constant A. F. García-Muñoz T. Neuman S. Neuman T. (2018). A “Healthy Immigrant Effect” or a “Sick Immigrant Effect”? Selection and Policies Matter. Eur. J. Health Econ. 19 (1), 103–121. 10.1007/s10198-017-0870-1 28144758

[B11] Constant A. F. (2021). “Return, Circular, and Onward Migration Decisions in a Knowledge Society,” in The Economic Geography of Cross-Border Migration. Editors Kourtit K. Newbold B. Nijkamp P. Patridge M. (New York, United States: Springer, Cham), 133–156. 10.1007/978-3-030-48291-6_7

[B13] Davidovitch N. Filc D. Novack L. Balicer R. D. (2013). Immigrating to a Universal Health Care System: Utilization of Hospital Services by Immigrants in Israel. Health Place. 20, 13–18. 10.1016/j.healthplace.2012.11.005 23291060

[B14] Deaton A. (2008). Income, Health, and Well-Being Around the World: Evidence From the Gallup World Poll. J. Econ. Perspect. 22 (2), 53–72. 10.1257/jep.22.2.53 19436768 PMC2680297

[B15] Donovan J. d'Espaignet C. M. van Ommeren M. (1992). Immigrants in Australia: A Health Profile. Canberra: AGPS.

[B16] Eikemo T. A. Huisman M. Bambra C. Kunst A. E. (2008). Health Inequalities According to Educational Level in Different Welfare Regimes: a Comparison of 23 European Countries. Sociol. Health Illn. 30 (4), 565–582. 10.1111/j.1467-9566.2007.01073.x 18298629

[B17] Feikin D. R. Nguyen L. M. Adazu K. Ombok M. Audi A. Slutsker L. (2009). The Impact of Distance of Residence From a Peripheral Health Facility on Pediatric Health Utilisation in Rural Western Kenya. Trop. Med. Int. Health. 14 (1), 54–61. 10.1111/j.1365-3156.2008.02193.x 19021892

[B18] Fortin N. Lemieux T. Firpo S. (2011). “Decomposition Methods in Economics,” in Handbook of Labor Economics. Editor Ashenfelter O. Cambridge MA, Vol. 4, 1–102. 10.1016/s0169-7218(11)00407-2

[B19] Harel Z. Kahana B. Kahana E. (1993). “Social Resources and the Mental Health of Aging Nazi Holocaust Survivors and Immigrants,” in International Handbook of Traumatic Stress Syndromes. Editors Wilson J. P. Raphael B. (Boston, MA: Springer), 241–252. 10.1007/978-1-4615-2820-3_20

[B20] Huisman M. Kunst A. E. Mackenbach J. P. (2003). Socioeconomic Inequalities in Morbidity Among the Elderly; a European Overview. Soc. Sci. Med. 57 (5), 861–873. 10.1016/S0277-9536(02)00454-9 12850111

[B21] Jasso G. Massey D. S. Rosenzweig M. R. Smith J. P. (2004). “Immigrant Health, Selectivity and Acculturation,” in Critical Perspectives on Racial and Ethnic Differences in Health in Late Life. Editors Anderson N. B. Bulatao R. A. Cohen B. (Washington, D.C.: National Academy Press), 227–266. 20669464

[B22] Kreft D. Doblhammer G. (2012). Contextual and Individual Determinants of Health Among Aussiedler and Native Germans. Health Place. 18 (5), 1046–1055. 10.1016/j.healthplace.2012.05.008 22784776

[B23] Ku L. Matani S. (2001). Left Out: Immigrants' Access to Health Care and Insurance. Health Aff. (Millwood). 20 (1), 247–256. 10.1377/hlthaff.20.1.247 11194848

[B24] LaVeist T. A. (2005). Disentangling Race and Socioeconomic Status: a Key to Understanding Health Inequalities. J. Urban Health. 82 (3), iii26–iii34. 10.1093/jurban/jti061 15933328 PMC3455905

[B50] Lennox C. S. Francis J. R. Wang Z. (2012). Selection Models in Accounting Research. Account. Rev. 87 (2), 589–616.

[B25] Manning W. G. Duan N. Rogers W. H. (1987). Monte Carlo Evidence on the Choice between Sample Selection and Two-Part Models. J. Econom. 35 (1), 59–82. 10.1016/0304-4076(87)90081-9

[B26] Maskileyson D. (2014). Healthcare System and the Wealth-Health Gradient: a Comparative Study of Older Populations in Six Countries. Soc. Sci. Med. 119, 18–26. 10.1016/j.socscimed.2014.08.013 25137644

[B27] Maskileyson D. Semyonov M. Davidov E. (2019). In Search of the Healthy Immigrant Effect in Four West European Countries. Soc. Incl. 7 (4), 304–319. 10.17645/si.v7i4.2330

[B28] Maskileyson D. Semyonov M. Davidov E. (2021). Economic Integration of First- and Second-Generation Immigrants in the Swiss Labour Market: Does the Reason for Immigration Make a Difference? Popul. Space Place. 27. 10.1002/psp.2426

[B29] McDonald J. T. Kennedy S. (2004). Insights Into the 'Healthy Immigrant Effect': Health Status and Health Service Use of Immigrants to Canada. Soc. Sci. Med. 59 (8), 1613–1627. 10.1016/j.socscimed.2004.02.004 15279920

[B30] Monheit A. C. Vistnes J. P. (2000). Race/Ethnicity and Health Insurance Status: 1987 and 1996. Med. Care Res. Rev. 57 Suppl 1 (1_Suppl. l), 11–35. 10.1177/1077558700057001S02 11092156

[B31] Nakash O. Nagar M. Shoshani A. Zubida H. Harper R. A. (2012). The Effect of Acculturation and Discrimination on Mental Health Symptoms and Risk Behaviors Among Adolescent Migrants in Israel. Cultur Divers. Ethnic Minor. Psychol. 18 (3), 228–238. 10.1037/a0027659 22686145

[B32] Neuman S. Oaxaca R. L. (2004). Wage Decompositions With Selectivity-Corrected Wage Equations: a Methodological Note. The J. Econ. Inequality. 2 (1), 3–10. 10.1023/b:joei.0000028395.38694.4b

[B33] Oaxaca R. (1973). Male-Female Wage Differentials in Urban Labor Markets. Int. Econ. Rev. 14, 693–709. 10.2307/2525981

[B51] O’Donnell O. A. Van Doorslaer E. K. A. Wagstaff A. (2006). “Decomposition of Inequalities in Health and Health Care,” in The Elgar Companion to Health Economics. Editor Jones A. M. (Cheltenham, United Kingdom: Edward Elgar). p. 179–192.

[B34] O’Donnell O. Van Doorslaer E. Wagstaff A. (2012). “Decomposition of Inequalities in Health and Health Care,” in The Elgar Companion to Health Economics. Editor Jones A.. Second Edition (Cheltenham, United Kingdom: Edward Elgar Publishing).

[B35] Padela A. I. Punekar I. R. (2009). Emergency Medical Practice: Advancing Cultural Competence and Reducing Health Care Disparities. Acad. Emerg. Med. 16 (1), 69–75. 10.1111/j.1553-2712.2008.00305.x 19055674

[B36] Palloni A. Arias E. (2004). Paradox Lost: Explaining the Hispanic Adult Mortality Advantage. Demography. 41 (3), 385–415. 10.1353/dem.2004.0024 15461007

[B37] Parkerson G. R. Jr Bridges-Webb C. Gervas J. Hofmans-Okkes I. Lamberts H. Froom J. (1996). Classification of Severity of Health Problems in Family/General Practice: an International Field Trial. Fam. Pract. 13 (3), 303–309. 10.1093/fampra/13.3.303 8671140

[B38] Parkerson G. R. Jr Broadhead W. E. Tse C. K. (1993). The Duke Severity of Illness Checklist (DUSOI) for Measurement of Severity and Comorbidity. J. Clin. Epidemiol. 46 (4), 379–393. 10.1016/0895-4356(93)90153-R 8483003

[B39] Ronellenfitsch U. Razum O. (2004). Deteriorating Health Satisfaction Among Immigrants from Eastern Europe to Germany. Int. J. Equity Health. 3 (1), 4. 10.1186/1475-9276-3-4 15193155 PMC441401

[B40] Saabneh A. (2015). Ethnic Health Inequalities in Unequal Societies: Morbidity Gaps between Palestinians and Jews in Israel. Eur. J. Popul. 31 (4), 445–466. 10.1007/s10680-015-9349-x

[B41] Semyonov M. Lewin-Epstein N. Bridges W. P. (2011). Explaining Racial Disparities in Access to Employment Benefits. Ethnic Racial Stud. 34 (12), 2069–2095. 10.1080/01419871003687552

[B42] Semyonov M. Lewin-Epstein N. Maskileyson D. (2013). Where Wealth Matters More for Health: the Wealth-Health Gradient in 16 Countries. Soc. Sci. Med. 81, 10–17. 10.1016/j.socscimed.2013.01.010 23422055

[B43] Semyonov M. Lewin-Epstein N. (2003). “Immigration and Ethnicity in Israel: Returning Diaspora and Nation-Building,” in Diasporas and Ethnic Migrants. Editors Rainer M. Rainer O. (London: Frank Cass), 327–337.

[B44] Semyonov M. Raijman R. Maskileyson D. (2015). Ethnicity and Labor Market Incorporation of Post-1990 Immigrants in Israel. Popul. Res. Pol. Rev. 34 (3), 331–359. 10.1007/s11113-014-9345-6

[B46] The Israeli Law of Return (1950). 4 L.S.I. 114 (1949–1950), Confers on Every Jew, with Some Minor Exceptions, the Right to Immigrate to Israel and Become an Israeli Citizen upon Arrival. New York, United States: Springer.

[B47] Williams D. R. Collins C. (2016). Racial Residential Segregation: a Fundamental Cause of Racial Disparities in Health. Public Health Rep. 116 (5), 404–416. 10.1093/phr/116.5.404 PMC1497358 12042604

[B48] Wolff J. L. Starfield B. Anderson G. (2002). Prevalence, Expenditures, and Complications of Multiple Chronic Conditions in the Elderly. Arch. Intern. Med. 162 (20), 2269–2276. 10.1001/archinte.162.20.2269 12418941

[B49] Zuvekas S. H. Taliaferro G. S. (2003). Pathways to Access: Health Insurance, the Health Care Delivery System, and Racial/Ethnic Disparities, 1996-1999. Health Aff. (Millwood). 22 (2), 139–153. 10.1377/hlthaff.22.2.139 12674417

