# Supplementary material for: Unhealthy Immigrants: Sources for Health Gaps Between Immigrants and Natives in Israel
Source: Front Sociol. 2021 Nov 1;6:686306. doi: 10.3389/fsoc.2021.686306 (PMC8591089; doi:10.3389/fsoc.2021.686306)
Supplement: Supplementary file 1 [file DataSheet1.PDF]

Supplementary Material

Appendix 1: Classification of severity of health problems

Illness	DUSOI diagnosis severity
Asthma	43.8
Hypertension	26.1
Cholesterol	17.1
Triglycerides	25.7
Heart attack	62.5
Angina pectoris	41.5
Congestive heart failure	48.7
Other heart disease	41.5
Stroke	62.5
Lung disease	53.2
Arthritis	50.0
Osteoporosis	48.8
Crohn's disease	42.9
Colitis	42.9
Cancer	62.5
Migraine	51.9
Anxiety	44.9
Depression	46.5
Thyroid disease	25.7
Diabetes	35.5

Note: Based on Duke Severity of Illness Checklist (DUSOI) (for details see Parkerson et al., 1996). As stroke, cancer and thyroid disease were not included in the DUSOI, stroke and cancer were assigned the same weight as a heart attack, and thyroid disease – the same weight as triglycerides.

Appendix 2: Definitions of the variables

Component	Variable	Measurement
Dependent	Severity illness index	in good health = 1; any physical health difficulties = 0
Demographics	Years since migration	In years
	Age of respondent	In years
	Marital status	Married = 1; Not married = 0
	Number of children	Number of children
Socioeconomic status	Education	In years
	Employment status	Employed = 1; unemployed or out of the labor market = 0
	Monthly household net income	Income less than 8,000 NIS = 1; Income higher than 12,000 NIS = 1; Missing income = 1; Income 8,000-12,000 NIS = 0
Health-related behavior	Fruits and vegetables consumption	Less than 1 vegetable/fruit portion per day = 1; More than 3 vegetable/fruit portions per day = 1; Missing for vegetable/fruits =1; 1-3 vegetable/fruit portions per day = 0
	Smoking	Current or former smoker = 1; never smoked = 0
	Sport	Participates in a physical activity = 1; does not participate in a physical activity = 0

Appendix 3: Decomposition of the total gap in the severity illness index (measured on a 100-point percentile scale) between Israeli native-born and immigrant groups, by gender

	Men				Women			
	**All Immigrants**	**FSU**	**EUAM**	**MENA**	**All Immigrants**	**FSU**	**EUAM**	**MENA**
**Total gap**	-27.901**	-40.904	-38.677***	-12.160	-33.555**	-36.504	-44.126**	-49.743***
Explained	-8.632***	-3.501**	-8.901*	-18.853***	-10.461***	-7.609***	-9.455**	-21.034***
Unexplained	-19.269	-37.403	-29.776**	6.693	-23.094	-28.895	-34.670*	-28.710*
Explained/Gross Gap, %	30.938	8.559	23.013	155.042	31.174	20.845	21.428	42.285
**Gap due to different source**	-8.632	-3.501	-8.901	-18.853	-10.461	-7.609	-9.455	-21.034
**Sources:**								
Demographics	-6.942**	-3.335**	-7.219*	-14.278***	-7.765**	-6.249**	-7.430**	-14.964***
Socioeconomic status	-1.338**	0.039	-1.147	-4.567***	-2.837***	-1.610***	-2.653***	-5.915***
Health behavior	-0.352	-0.205	-0.535	-0.008	0.142	0.250	0.628	-0.155

Note: Note: Standard errors in parentheses; *** p < 0.001, ** p < 0.01, * p < 0.05. All calculations based on a two-stage estimation procedure using the inverse Mills ratio to correct for the selection bias. Adjusted coefficients are presented.
